# A Dogma in Doubt: Hydrolysis of Equatorial Ligands of Pt^IV^ Complexes under Physiological Conditions

**DOI:** 10.1002/anie.201900682

**Published:** 2019-04-25

**Authors:** Alexander Kastner, Isabella Poetsch, Josef Mayr, Jaroslav V. Burda, Alexander Roller, Petra Heffeter, Bernhard K. Keppler, Christian R. Kowol

**Affiliations:** ^1^ University of Vienna Faculty of Chemistry Institute of Inorganic Chemistry Waehringer Strasse 42 1090 Vienna Austria; ^2^ Institute of Cancer Research and Comprehensive Cancer Center Medical University of Vienna Borschkegasse 8a 1090 Vienna Austria; ^3^ Department of Chemical Physics and Optics Faculty of Mathematics and Physics Charles University Ke Karlovu 3 12116 Prague 2 Czech Republic; ^4^ Research Cluster “Translational Cancer Therapy Research” Vienna Austria

**Keywords:** antitumor agents, hydrolysis, platinum(IV) complexes, prodrugs, reduction

## Abstract

Due to their high kinetic inertness and consequently reduced side reactions with biomolecules, Pt^IV^ complexes are considered to define the future of anticancer platinum drugs. The aqueous stability of a series of biscarboxylato Pt^IV^ complexes was studied under physiologically relevant conditions. Unexpectedly and in contrast to the current chemical understanding, especially oxaliplatin and satraplatin complexes underwent fast hydrolysis in equatorial position (even in cell culture medium and serum). Notably, the resulting hydrolysis products strongly differ in their reduction kinetics, a crucial parameter for the activation of Pt^IV^ drugs, which also changes the anticancer potential of the compounds in cell culture. The discovery that intact Pt^IV^ complexes can hydrolyze at equatorial position contradicts the dogma on the general kinetic inertness of Pt^IV^ compounds and needs to be considered in the screening and design for novel platinum‐based anticancer drugs.

Pt^II^ complexes still play a very important role in cancer treatment,^1^ contributing to about 50 % of all chemotherapies.^2^ Cis‐, carbo‐, and oxaliplatin are widely used against various forms of cancer.^3^ Furthermore, very recent clinical data show impressive synergistic effects of platinum drugs together with checkpoint inhibitor immunotherapy.^4^ The mode of action of these metal complexes involves aquation, binding to DNA, and subsequently induced apoptosis.^5^ However, Pt^II^ complexes bind to DNA not only in the tumor tissue, but also in healthy cells, resulting in (severe) side effects like nephro‐, neuro‐, and gastrointestinal‐tract toxicity.^6^ To reduce these adverse effects, Pt^IV^ complexes are attracting increasing interest.^7^ Such low‐spin d^6^ octahedral complexes are considered to be kinetically more inert^8^ and consequently far less reactive towards biomolecules.^9^ Activation by reduction to the active Pt^II^ complexes can occur via low‐molecular‐weight compounds like ascorbate and glutathione,^10^ and/or high‐molecular‐weight proteins.^11^ Another advantage of Pt^IV^ complexes are the two additional axial ligands, which can be used to optimize chemical and biological properties, like lipophilicity and reduction potential. Furthermore, bioactive substances or drug‐targeting moieties can be used as axial ligands.8b, 12 Despite these benefits, no Pt^IV^ drug has been clinically approved so far.^2^ Nevertheless, some candidates have entered clinical trials. However, Tetraplatin, ctc‐[Pt(DACH)Cl_4_] (DACH=(1*R*,2*R*)‐(−)‐1,2‐diaminocyclohexane), was discontinued because of its neurotoxicity,^13^ while Iproplatin, ctc‐[Pt(IPA)_2_(OH)_2_Cl_2_] (IPA=isopropylamine), did not show superior anticancer activity.^14^ The most prominent representative Satraplatin, ctc‐[Pt(NH_3_)(CHA)(OAc)_2_Cl_2_] (CHA=cyclohexylamine), ultimately failed to show improved overall survival in a phase III study.^2^ Nevertheless, current research strongly focuses on Pt^IV^ complexes, as they are considered to be the next generation of platinum‐based anticancer drugs.^7^ Consequently, the underlying chemical properties and reactivities are of high importance for the specific design of novel drugs with optimized biological properties. As already mentioned above, Pt^IV^ complexes are considered to be kinetically inert. However, there are a few reports that the axial ligands can be hydrolyzed when electron‐withdrawing ligands like dichloroacetate are present.^15^ Concerning the equatorial ligands of Pt^IV^ complexes, a study using plasma from patients treated with Satraplatin showed not only the parent Pt^II^ drug JM118, but unexpectedly also the mono‐ and dihydrated Pt^IV^ species, where one or two equatorial chlorido ligands were exchanged with hydroxido ligands.^16^ This is in strong contrast to the “dogma” of kinetic inertness of Pt^IV^ drugs. As such a hydrolysis would strongly impact the chemical characteristics (e.g. reduction potential) and consequently also the biological properties, we investigated in this study the hydrolysis of the equatorial ligands for a panel of different Pt^IV^ complexes in comparison to Satraplatin (Figure 1).

**Figure 1:**
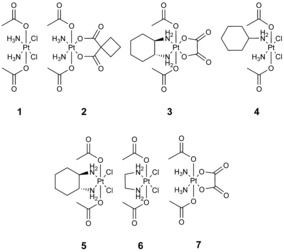
Investigated Pt^IV^ compounds.

To get a first overview, we analyzed the hydrolytic stability of the model complexes **1**–**3** and satraplatin **4** (Figure 1) under physiologically relevant conditions. All complexes possess two acetato ligands in axial position and an equatorial core consisting of cisplatin (**1**), carboplatin (**2**), oxaliplatin (**3**), or *cis*‐amminedichlorido(cyclohexylamine) (**4**). The complexes were incubated in phosphate buffer (PB) at 37 °C and pH 7.4 for 24 h and monitored using HPLC‐MS. Indeed, after 24 h most of the satraplatin (72 % hydroxido and 3 % dihydroxido species) and the oxaliplatin‐based complex **3** (43 % hydroxido and 14 % dihydroxido; Figure 2) was already hydrolyzed. In contrast, the cisplatin analogue **1** was widely stable (5 % hydroxido species) and the carboplatin derivative remained completely unchanged (Figure S1). To exclude a significant impact of the column on the hydrolysis process, the incubated solutions were also directly injected into the mass spectrometer. This revealed the same hydrolyzed products as observed with HPLC‐MS (data not shown).

**Figure 2:**
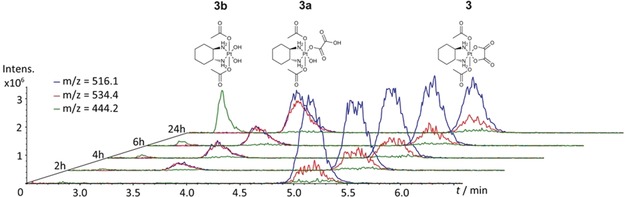
Time‐dependent hydrolysis of **3** (50 μm in PB) at 37 °C and pH 7.4 with formation of the hydroxido (**3 a**) and dihydroxido (**3 b**) complex.

Notably, repetition of these experiments at pH values of 8.0 and 9.0 even resulted in distinctly accelerated hydrolysis (Table 1). At pH 9.0 the parental oxaliplatin derivative **3** completely disappeared with exclusive formation of the dihydroxido species. Also satraplatin was fully converted into its hydrolyzed species (25 % hydroxido/75 % dihydroxido). In contrast, in the case of the cisplatin analogue **1**, still ≈70 % of the intact complex could be observed together with ≈20 % hydroxido and 5 % dihydroxido species. Carboplatin was again completely unaffected. These findings indicated a distinct pH‐dependency of the reaction, which could be exemplarily proven for **3**, which displayed a perfect linear correlation between pH and the reaction rate (Figure S2).

**Table 1:** Relative amount in % of the respective hydrolysis products of complexes **1**–**4** determined by HPLC‐MS after incubation of a 50 μm solution of the compound at 37 °C for 24 h in PB at different pH values.

		Compound			
pH	Species	**1**	**2**	**3**	**4**
7.4	% hydroxido	5	–	43	72
	% dihydroxido	–	–	14	3
8.0	% hydroxido	10	–	32	76
	% dihydroxido	2	–	53	19
9.0	% hydroxido	22	–	–	25
	% dihydroxido	5	–	100	75

To rule out the involvement of phosphate in the hydrolysis, other buffer systems, that is, HEPES and ammonium carbonate, were investigated for **3** yielding comparable results (Figure S3). Next, **3** was investigated in cell culture medium (RPMI‐1640), and similar ratios of hydroxido/dihydroxido complex formation were observed as in pure PB solution (Figure S3). Notably, the pH value of fresh cell culture medium was about 7.4 but increased up to pH 8–9 within 24 h. Consequently, the cell culture medium was phosphate‐buffered (150 mm), generating stable pH values over >24 h. Finally, the hydrolysis of **3** was also investigated in mouse serum (also buffered with 150 mm phosphate), again yielding similar results (Figure S3). This clearly indicates that all the components of cell culture medium or serum (amino acids, proteins, inorganic salts, and vitamins) have no influence on the hydrolysis process of the Pt^IV^ complex. Instead this reaction is solely dependent on the presence of water as a solvent and the appropriate pH value.

Notably, the hydrolysis of oxaliplatin with ≈10 %^17^ is distinctly slower than that of **3** with ≈50 % after 24 h at pH 7.4. A likely explanation is that the Pt^II^ complex with a monodentate oxalate ligand possesses a p*K*_a_ of 7.23^18^ and therefore an aqua ligand is partially present. This enables a fast ring‐closing reverse reaction to the bidentate‐bound oxalate and reformation of oxaliplatin. Consequently, the release of oxalate is suppressed. In contrast, the p*K*_a_ of the Pt^IV^ complex **3 a** is much lower at 3.5 (see below). Therefore a hydroxido ligand is still present at pH 7.4 and the reverse reaction back to **3** is hindered. This results in a shift of the equilibrium towards the dihydroxido species and as a consequence the hydrolysis is faster than in the case of Pt^II^.

To investigate whether the hydrolysis of **3** takes place via an attack on the electrophilic carboxylic group of the oxalate ligand or on platinum itself, we analyzed the hydrolysis process in H_2_^18^O (buffered with 50 mm phosphate). This measurement resulted in an exact mass of *m*/*z*=488 for ctc‐[Pt(DACH)(OAc)_2_(^18^OH)_2_]+Na^+^, which proves that the platinum core is directly attacked and not the oxalato ligand (Figure S4). This is in line with H_2_^18^O studies of the hydrolysis of the axial dichloroacetate ligands of mitaplatin.^15^

To gain more insight on the exact impact of the different equatorial ligands, the additional Pt^IV^ model complexes **5**–**7** (Figure 1) were synthesized. Table 2 presents an overview on the speed of hydrolysis of all complexes **1**–**7** after incubation in PB at pH 7.4 and 37 °C for 24 h. The following trends could be observed: 1) When the derivatives with a chlorido ligand as a leaving group are compared, the oxaliplatin‐like complex (DACH; **5**) has the fastest hydrolysis with complete disappearance of the parental compound after 24 h, followed by satraplatin (NH_3_/CHA; **4**), the ethylenediamine complex (en; **6**), and the cisplatin derivative (NH_3_; **1**) with just 5 % hydrolysis. 2) Comparison of the complexes with two NH_3_ equatorial ligands shows generally very slow hydrolysis rates with the cisplatin derivative **1** as the fastest (5 % hydrolysis), followed by the completely stable oxalate‐bearing complex **7** and the carboplatin analogue **2**. 3) When complexes with the same amine ligand but chlorido vs. oxalato leaving groups (e.g. **5** vs. **3**) are compared, the complex with chlorido ligands hydrolyzes faster (81 % hydroxido for **5**) than the one with the bidentate oxalate ligand (41 % for **3**). However, when the DACH ligand in complexes **5** and **3** is exchanged for two NH_3_ moieties, the hydrolytic stability dramatically increases, with only 5 % hydroxide species for **1** and no hydrolysis for **7**. This impressively shows that the influence of the leaving group (chlorido vs. oxalate) has much less impact on the hydrolysis compared to the stable amine ligand (DACH vs. NH_3_). Notably, incubation of **1**–**7** in cell culture medium (RPMI‐1640) resulted in similar amounts of hydrolysis products. As RPMI‐1640 contains ≈100 mm NaCl, this also confirms that the presence of chloride ions cannot significantly change the rate of hydrolysis (this could be also supported by comparison of **4**, **5**, and **6** in 50 mm PB vs. 50 mm PBS with 150 mm NaCl).

**Table 2:** Overview on the impact of equatorial ligands on the rate of hydrolysis in terms of % of complexes **1**–**7** determined by HPLC‐MS of a 50 μm solution of the compound after 24 h incubation in PB at 37 °C.

Complex	% Hydroxido	% Dihydroxido	Stable ligand^[b]^	Labile ligand
**1**	5	0	NH_3_	Cl
**2**	0	0	NH_3_	CBDCA
**3**	41	13	DACH	oxalate
**4**	72^[a]^	3	NH_3_/CHA	Cl
**5**	81	18	DACH	Cl
**6**	42	0	en	Cl
**7**	0	0	NH_3_	oxalate

[a] Two different monohydroxido species can be observed with a ratio of ≈1:6. [b] DACH=(1*R*,2*R*)‐(−)‐1,2‐diaminocyclohexane; CHA=cyclohexylamine; en=ethylenediamine; CBDCA=1,1‐cyclobutanedicarboxylic acid.

In order to obtain deeper insight into the underlying binding energies (BE) and heights of the activation barriers, a set of quantum‐chemical data was calculated. The BE obtained from energy decomposition of the individual reactants (Table S1) are in good agreement with the trends mentioned above. For example: comparison of the BE of the leaving chlorido ligands resulted also in the order **5**≈**4**>**6**>**1** (in the case of **4** the weaker‐bound chlorido ligand was considered). Furthermore, BE of the ammine ligands in complex **1**, **2**, and **7** were in line with the experimental data, although the BE of the acetato ligands are quite different. These results were also verified by extended transition state combined with natural orbitals for chemical valence (ETS‐NOCV)^19^ analysis and average local ionization potential (ALIP) maps.^20^ Additionally, the calculated heights of activation barrier of the hydrolysis (Scheme S1) of the equatorial ligands correlate very well with the HPLC‐MS experiments (Table 1). As summarized in Table 3, the activation energies for the hydrolysis in the case of **3** and **4** are distinctly lower than those for **1** and **2**. This confirms that the higher σ‐donor properties of a secondary amine compared to simple NH_3_ ligands are important for an accelerated reaction, which explains the differences observed in the HPLC‐MS experiments.^21^ Furthermore, also the second hydrolysis step Δ*E*(TS2) of **3**, where the monodentate‐bound oxalate ligand is finally released, possesses a distinctly lower activation barrier than that of **4** with two chlorido ligands; this is in exact agreement with the HPLC‐MS studies (Table 1). The calculations in the case of **4** further revealed a higher *trans*‐effect of the CHA ligand compared to NH_3_ explaining the two peaks observed with HPLC‐MS. This is in accordance with a report on the respective Pt^II^ complex JM118.^22^

**Table 3:** Activation energies (in kcal mol^−1^) for replacement of the first (Δ*E*(TS1)) and the second (Δ*E*(TS2)) equatorial leaving group.

Complex	**1**	**2**	**3**	**4** ^[a]^	**4** ^[b]^
Δ*E*(TS1)	31.1	31.6	27.0	25.7	26.1
Δ*E*(TS2)	27.3	30.4	22.6	24.8	25.0

[a] *trans* to CHA. [b] *trans* to NH_3_.

To test how the hydrolysis alters the chemical and biological properties, the two derivatives of **3** were synthesized. This was achieved through incubation of **3** at pH 8–9 and 37 °C and subsequent purification via preparative HPLC. The hydroxido (**3 a**) and the dihydroxido (**3 b**) species were characterized by ^1^H and ^13^C NMR, mass spectrometry, and elemental analysis. Furthermore, the p*K*_a_ values of **3 a** and **3 b** were determined via ^1^H NMR analysis and found to be 3.5 and 4.0, respectively (Figure 3).

**Figure 3:**
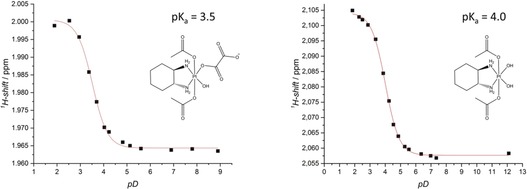
Determination of p*K*_a_ values via correlation between the chemical shift (in ppm) of the acetato ligand and pD for **3 a** (left) and **3 b** (right).

This is in line with data of **3 a** after ≈3 h of incubation at pH of 2.5, which resulted in a complete transformation back to its parental complex **3**. According to the p*K*_a_ value, the hydroxido ligand gets protonated at such low pH values and the thereby generated aqua ligand can be released under reformation of the bidentate oxalato complex **3**. In contrast, **3 a** is stable at pH 5.5, which again fits with the p*K*_a_ value of 3.5.

After the incubation of **3 b** in MeOH/Et_2_O a few single crystals could be obtained and were analyzed by X‐ray diffraction. The structure reveals an octahedral geometry with two axial acetato ligands and the equatorial DACH moiety. However, the two hydroxido groups were exchanged by methoxido ligands (Figure 4; for bond lengths and angles see Tables S5 and S6) Notably, the crystals contain both the *R*,*R* and *S*,*S* isomers as a racemic mixture. This can be explained by the ≈2 % *S*,*S* isomer present in the commercially available DACH compound and the often observed preference of compounds to crystallize as a racemate and not as the pure isomers.^23^

**Figure 4:**
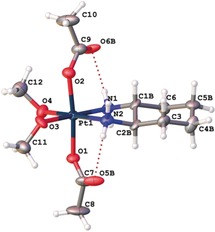
X‐ray crystal structure of **3 b** incubated in MeOH/Et_2_O (the disorder of the DACH ligand is not shown).

As a next step, the reactivity of **3 b** with different organic solvents was investigated. In contrast to aqueous cell culture medium or serum, incubation of **3 b** with, for example, DMSO, acetonitrile, MeOH, or EtOH for 1 h resulted in the exchange of one hydroxido ligand, which could be proven by mass spectrometry and an altered HPLC retention time (Figure S5). This indicates that at very high excess, the hydroxido ligands indeed can be substituted, which could also be used as a new synthetic pathway for introducing equatorial ligands into already existing Pt^IV^ complexes.

As a next step, the thermodynamic reduction properties of **3**, **3 a**, and **3 b** were compared using cyclic voltammetry. All three complexes showed irreversible reduction peaks with decreasing potentials the more hydroxido ligands are present in the molecule (**3**: −630 mV vs. NHE; **3 a**: −670 mV vs. NHE; **3 b**: −920 mV vs. NHE). This trend is in line with data from similar Pt^IV^ complexes, however, with one or two axial hydroxido groups.^24^ The kinetic reduction rates of **3**, **3 a,** and **3 b** were investigated by HPLC after incubation with 10 equiv. of l‐ascorbic acid at 20 °C. While **3** was completely stable over 6 h, **3 a** and **3 b** were reduced much faster and fully converted to the respective Pt^II^ species already after 3–4 h (Figure 5). Consequently, these hydroxide species are even more rapidly reduced than the cisplatin complex **1**, which is well‐known to be much more sensitive than oxaliplatin or carboplatin derivatives.^25^ Thus, although the thermodynamic reduction potential decreases with the increasing number of OH groups, the reduction rate accelerates dramatically. Although this seems to be unexpected, these data are in line with a study of Gibson et al.^24^ using axial mono‐ and dihydroxido derivatives of complex **3** and support the importance of the Pt^IV^ reduction kinetics.

**Figure 5:**
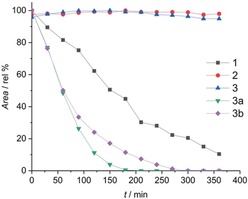
Reduction rate of 1 mm **1**, **2**, **3**, **3 a**, and **3 b** at 20 °C with 10 equiv. l‐ascorbic acid in 250 mm phosphate buffer at pH 7.4 monitored by HPLC.

To evaluate whether the changed chemical properties of the hydrolysis products result in differences in biological activity, the anticancer activity of **3**, **3 a**, and **3 b** against three cancer cell lines (HCT116, RKO, and CT‐26) was evaluated. These experiments revealed that **3 b** had a significantly lower IC_50_ value (up to 2‐fold more active) than the parental species **3** or the monohydroxido species **3 a** (Figure 6 and Figure S6; Table 4).

**Figure 6:**
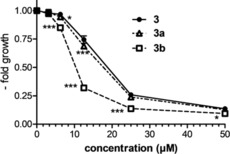
Anticancer activity of **3**, **3 a**, and **3 b** after 72 h against HCT116 cells measured by MTT assay. The values given are means ± standard deviation of one representative experiment performed in triplicate. * *p*<0.05, *** *p*<0.001.

**Table 4:** IC_50_ values of **3**, **3 a**, and **3 b** against cancer cells after 72 h exposure. Values represent mean ± standard deviation (SD) from three or four biologically independent experiments performed in triplicate.

Cell line	**3**		**3 a**		**3 b**	
	IC_50_ [μm]	±SD	IC_50_ [μm]	±SD	IC_50_ [μm]	±SD
HCT116	16.2	±2.0	16.6	±2.4	11.8	±2.1
RKO	12.5	±2.8	15.6	±4.9	9.4	±2.9
CT‐26	18.7	±2.2	13.7	±1.8	8.2	±2.1

An explanation for this could be that after reduction of **3 a,** the hydroxido group in the respective Pt^II^ complex is protonated (p*K*_a_=7.23).^18^ This aqua ligand represents a good leaving group and facilitates ring closure to the bidentate‐bound oxalate and reformation of oxaliplatin. Consequently, **3** and **3 a** can be expected to have a very similar cytotoxic activity. In contrast, in the case of **3 b**, oxaliplatin cannot be regenerated, and instead the Pt^II^ complex [Pt(DACH)(H_2_O)OH]^+^ is formed, which is able to directly interact with biological targets. In addition, cellular uptake of **3**, **3 a**, and **3 b** was studied on HCT‐116 and CT‐26 cells after 3 h incubation. Notably, no significant differences in the uptake could be observed, with the parental complex **3** showing the highest cellular platinum levels (Figure S7).

Taken together, our data indicates that we have to reconsider the current understanding of anticancer Pt^IV^ complexes and doubt the dogma of the generally very high hydrolytic stability of Pt^IV^ complexes. Depending on the exact equatorial coordination sphere, there are massive differences in their stability at physiological pH, and the resulting hydrolyzed Pt^IV^ complexes possess vastly altered physicochemical properties. Especially their rate of reduction, the crucial factor in the activation of Pt^IV^ prodrugs, is strongly accelerated upon prior hydrolysis. These observations are also important for, for example, simple physiologically buffered solutions of oxaliplatin(IV) complexes, where more than 50 % hydrolysis can be expected within 24 h. Furthermore, not the leaving group itself, but the substituents at the equatorial amine ligands have the major impact on the rate of hydrolysis. In addition, it is important to mention that (cancer) cells possess different pH values in their organelles, for example, up to pH 8 in mitochondria.^26^ Consequently, even higher levels of hydrolysis can be expected, as this reaction is further accelerated at more alkaline pH values. Under such conditions also cisplatin derivatives start to hydrolyze to a significant degree.

Thus, it is essential to carefully select the equatorial core of Pt^IV^ prodrugs not only according to the biological activity of the active Pt^II^ analogue, but also to the hydrolytic stability of the Pt^IV^ prodrugs (satraplatin<oxaliplatin≪cisplatin<carboplatin) and the rate of reduction (satraplatin>cisplatin≫oxaliplatin≈carboplatin). All these parameters influence the biological properties and have to be considered in the future design of novel Pt^IV^ anticancer prodrugs.

## Conflict of interest

The authors declare no conflict of interest.

## Supporting information

As a service to our authors and readers, this journal provides supporting information supplied by the authors. Such materials are peer reviewed and may be re‐organized for online delivery, but are not copy‐edited or typeset. Technical support issues arising from supporting information (other than missing files) should be addressed to the authors.

Supplementary
