# Supplementary material for: A Dogma in Doubt: Hydrolysis of Equatorial Ligands of PtIV Complexes under Physiological Conditions
Source: Angew Chem Int Ed Engl. 2019 Apr 25;58(22):7464–9. doi: 10.1002/anie.201900682 (PMC6766845; doi:10.1002/anie.201900682)
Supplement: Supplementary file 1 — Supplementary [file ANIE-58-7464-s001.pdf]

Angewandte Chemie
Eine Zeitschrift der Gesellschaft Deutscher Chemiker

Supporting Information

A Dogma in Doubt: Hydrolysis of Equatorial Ligands of Pt^{IV} Complexes under Physiological Conditions

*Alexander Kastner, Isabella Poetsch, Josef Mayr, Jaroslav V. Burda, Alexander Roller, Petra Heffeter, Bernhard K. Keppler, and Christian R. Kowol**

anie_201900682_sm_miscellaneous_information.pdf

Chemicals and Instrumentation

Potassium tetrachloridoplatinate ($K_2[PtCl_4]$) was purchased from Johnson Matthey (Switzerland). Satraplatin (**4**) was bought from BOC Sciences (USA). Water for synthesis was taken from a reverse osmosis system and distilled twice before use. For HPLC measurements Milli-Q water (18.2 MΩ·cm, Merck Milli-Q Advantage, Darmstadt, Germany) was used. Other chemicals and solvents were purchased from commercial suppliers (Sigma Aldrich, Merck and Fisher Scientific). Electrospray ionization (ESI) mass spectra were recorded on a Bruker amaZon SL ion trap mass spectrometer in positive and/or negative mode by direct infusion. High resolution mass spectra were measured on a Bruker maXis™ UHR ESI time of flight mass spectrometer. One- and two-dimensional ^{1}H-NMR and ^{13}C-NMR spectra were recorded on a Bruker Avance III 500 MHz spectrometer at 500.10 (^{1}H) MHz at 298 K. For ^{1}H-NMR spectra the solvent residual peak was taken as internal reference. Elemental analysis measurements were performed on a Perkin Elmer 2400 CHN Elemental Analyzer at the Microanalytical Laboratory of the University of Vienna.

Synthesis of Complexes

The platinum(II) complexes for **1-3** and **5-7** were synthesized according to literature.[1–4]

The next two reaction steps were similar for all synthesized compounds. The general procedures were as follows:

Synthesis of the dihydroxidoplatinum(IV) complexes. To a solution of the platinum(II) complex in tri-distilled water (0.1 mM), H_2O_2 (50 %, 20 eq) was added and the solution was stirred in the dark for 26 h. The solvent was removed, the residue was taken up as a suspension in methanol and fully precipitated by addition of diethyl ether. The product was filtered off and used without further purification.

Synthesis of the diacetatoplatinum(IV) complexes. A suspension of the dihydroxidoplatinum(IV) complex in acetic anhydride (0.1 mM) was stirred for 120 h in the dark. Subsequently, diethyl ether was added to fully precipitate the raw product, which was then filtered off and washed with diethyl ether. The raw product was purified via preparative RP-HPLC.

Compound 1

(*OC*-6-33)-Diacetatodiamminedichloridoplatinum(IV).[5] HPLC-conditions: isocratic with 16% acetonitrile; Yield: 15% as a white solid; ^{1}H-NMR (DMSO-d$_6$): δ = 6.54 (m, 6H, N*H*$_3$), 1.91 (s, 6H, C*H*$_3$); MS: calcd. for $[C_4H_{12}Cl_2N_2O_4Pt\text{-}Na^+]^+$: 440.97, found: 441.01.

Compound 2

(*OC*-6-33)-Diacetatodiammine(1,1-cyclobutanedicarboxylato)platinum(IV).
HPLC-conditions: gradient of 1-16% acetonitrile in 18 min; Yield: 20% as a white solid; ^{1}H-NMR (DMSO-d$_6$): δ = 6.37 (m, 6H, N*H*$_3$), 2.48 (under solvent peak, 4H, CC*H*$_2$CH$_2$), 1.90 (s, 6H, C*H*$_3$), 1.82 (qui, J = 8.0, 2H, CH$_2$C*H*$_2$CH$_2$); MS: calcd. for $[C_{10}H_{18}N_2O_8Pt\text{-}Na^+]^+$: 512.0609, found: 512.0587. elemental analysis calcd. for $C_{10}H_{18}N_2O_8Pt$: C: 24.54, H: 3.71, N: 5.72, found: C: 27.47, H: 3.45, N: 5.65

Compound 3

(*OC*-6-33)-Diacetato[(1*R*,2*R*)-cyclohexane-1,2-diamine]oxalatoplatinum(IV).[6]
HPLC-conditions: isocratic with 5% acetonitrile; Yield: 46% as a white solid; ^{1}H-NMR (DMSO-d$_6$): δ = 8.38 (bs, 2H, N*H*$_2$), 8.30 (bs, 2H, N*H*$_2$), 2.56 (bs, 2H, C*H*$_{dach}$), 2.11 (m, 2H, C*H*$_{2,dach}$), 1.96 (s, 6H, C*H*$_3$), 1.51-1.41 (m, 4H, C*H*$_{2,dach}$), 1.16 (m, 2H, C*H*$_{2,dach}$); MS: calcd. for $[C_{12}H_{20}N_2O_8Pt\text{-}Na^+]^+$: 538.08, found: 538.09.

Compound 5

(*OC*-6-33)-Diacetatodichlorido[(1*R*,2*R*)-cyclohexane-1,2-diamine]platinum(IV). [7]
HPLC-conditions: isocratic with 5% acetonitrile; Yield: 54% as a white/yellow solid; ^{1}H-NMR (DMSO-d$_6$): δ = 9.44 (bs, 2H, N*H*$_2$), 8.25 (bs, 2H, N*H*$_2$), 2.56 (bs, 2H, C*H*$_{dach}$), 2.19 (m, 2H, C*H*$_{2,dach}$), 1.95 (s, 6H, C*H*$_3$), 1.52 (m, 2H, C*H*$_{2,dach}$), 1.33 (m, 2H, C*H*$_{2,dach}$), 1.14 (m, 2H, C*H*$_{2,dach}$); MS: calcd. for $[C_{10}H_{20}Cl_2N_2O_4Pt\text{-}Na^+]^+$: 521.03, found: 521.12.

Compound 6

(*OC*-6-33)-Diacetatodichlorido(ethylenediamine)platinum(IV).[8] HPLC-conditions: Atlantis T3 Prep Column, gradient of 1-10% acetonitrile in 15 min; Yield: 38% as a white solid; ^{1}H-NMR (DMSO-d_6): δ = 8.54 (bs, 4H, N*H*$_2$), 2.65 (m, 4H, C*H*$_2$), 1.94 (s, 6H, C*H*$_3$); MS: calcd. for [$C_6H_{14}Cl_2N_2O_4$Pt-Na^+]$^+$: 465.9871, found: 465.9860.

Compound 7

(*OC*-6-33)-Diacetatodiammineoxalatoplatinum(IV). HPLC-conditions: Atlantis T3 Prep Column, gradient of 1-15% acetonitrile in 15 min; Yield: 33% as a white solid; ^{1}H-NMR (DMSO-d_6): δ = 6.52 (m, 6H, N*H*$_3$), 1.94 (s, 6H, C*H*$_3$); MS: calcd. for [$C_6H_{12}N_2O_8$Pt-Na^+]$^+$: 458.0139, found: 458.0130; elemental analysis calcd. for $C_6H_{12}N_2O_8$Pt: C: 16.56, H: 2.78, N: 6.44 found: C: 16.56, H: 2.805, N: 6.36.

Compound 3a

(*OC*-6-32)-Diacetato[(1*R*,2*R*)-cyclohexane-1,2-diamine]hydroxidooxalatoplatinum(IV). Compound **3** (50 mg, 0.097 mmol) was dissolved in 50 mM phosphate buffer (40 ml) at pH 8 and incubated at 37°C for 24 hours. The resulting purple-brown solution was lyophilized and afterwards purified via preparative HPLC. HPLC-conditions: Atlantis T3 Prep Column, H_2O/ACN, gradient of 1-15% acetonitrile in 15 min; Yield: 12.1 mg (23.4%) as a; ^{1}H-NMR (D_2O): δ = 2.76 (dt, 1H, J = 17.35 Hz, J = 3.82 Hz, C*H*$_{dach}$), 2.71 (dt, 1H, J = 11.56 Hz, J = 3.75 Hz, C*H*$_{dach}$), 2.24 (t, 2H, J = 16.84, C*H*$_{2,dach}$), 2.06 (s, 3H, C*H*$_3$), 2.05 (s, 3H, C*H*$_3$), 1.61 (m, 2H, C*H*$_{2,dach}$), 1.48 (t, 2H, J = 11.95, C*H*$_{2,dach}$), 1.19 (m, 2H, C*H*$_{2,dach}$); ^{13}C-NMR (D_2O): δ = 182.83 (CH_3*C*OO), 182.63 (CH_3*C*OO), 171.76 (COO*C*OOH), 166.57 (*C*OOCOOH), 61.79 (*C*H$_{dach}$), 61.45 (*C*H$_{dach}$), 31.45 (*C*H$_{2,dach}$), 30.68 (*C*H$_{2,dach}$), 23.48 (*C*H$_{2,dach}$), 22.25 (*C*H$_3$), 22.08 (*C*H$_3$); MS: calcd. for [$C_{12}H_{22}N_2O_9$Pt-Na^+]$^+$: 556.0867, found: 556.0849; elemental analysis calcd. for $C_{12}H_{22}N_2O_9$Pt·3 H_2O: C: 24.53, H: 4.80, N: 4.77, found: C: 23.89, H: 3.85, N: 4.66.

Compound 3b

(*OC*-6-13)-Diacetato[(1*R*,2*R*)-cyclohexane-1,2-diamine]dihydroxidoplatinum(IV).

Compound **3** (50 mg, 0.097 mmol) was dissolved in 40 ml 50 mM phosphate buffer at pH 9 and incubated at 37°C for 24 hours. The resulting purple-brown solution was lyophilized and afterwards purified via preparative HPLC. HPLC-conditions: Atlantis T3 Prep Column, H_2O/ACN, gradient of 1-15% acetonitrile in 15 min; Yield: 32.1 mg (71.7%) as a yellow solid (quite hydroscopic); ^{1}H-NMR (D_2O): δ = 2.71 (m, 2H, CH_{dach}), 2.18 (m, 2H, $CH_{2,dach}$), 2.06 (s, 6H, CH_3), 1.61 (m, 2H, $CH_{2,dach}$), 1.45 (m, 2H, $CH_{2,dach}$), 1.18 (m, 2H, $CH_{2,dach}$); ^{13}C-NMR (D_2O): δ = 182.79 (CH_3COO), 61.35 (CH_{dach}), 31.51 ($CH_{2,dach}$), 23.58 ($CH_{2,dach}$), 22.50 (CH_3); MS: calcd. for $[C_{10}H_{22}N_2O_6Pt\text{-}Na^+]^+$: 484.1019, found: 484.1008; elemental analysis calcd. for $C_{10}H_{22}N_2O_6Pt \cdot H_2O$: C: 25.05, H: 5.05, N: 5.84, found: C: 25.37, H: 5.02, N: 5.83.

HPLC-MS-System

All hydrolysis experiments were monitored on an Agilent 1260 Infinity system using a Waters Atlantis T3 column 150 mm x 4.6 mm coupled to a Bruker amaZon SL ESI-IT mass spectrometer. Milli-Q water, containing 0.1% formic acid, and acetonitrile containing 0.1% formic acid were used as eluents. A gradient of 1-40% acetonitrile in 20 min was used.

Purification by preparative RP-HPLC

The compounds were purified by preparative RP-HPLC using a Waters XBridge C18 column or a Waters Atlantis T3 column on an Agilent 1200 Series system. Milli-Q water, containing 0.1% formic acid, and acetonitrile were used as eluents unless otherwise stated.

Extraction of platinum compounds from serum

For method validation, 18 µl mouse serum were spiked with a known ratio of **3**, **3a** and **3b** in 2 µl phosphate buffer (50 mM, pH 7.4) with an overall platinum concentration of 500 µM. Immediately afterwards the platinum species were extracted via addition of different amounts of acetonitrile or methanol in order to test different methods. After vigorously shaking for 1 min the suspension was centrifuged at 6000 rpm for 10 min. The supernatant was taken up with a syringe leaving a pellet of denaturized proteins behind and was directly measured via HPLC-MS. Furthermore, 10 kDa centrifugal filters were tested for their efficacy. The spiked serum sample was put inside and the sample was centrifuged at 6000 rpm for 10 min and the filtrate directly injected into HPLC-MS. These measurements proved the extraction via the addition of

methanol, in a serum to methanol ratio of 1:3, to be the most effective. It showed a recovery of 88.5%, as well as the initial peak area ratio for **3**, **3a** and **3b**.

For kinetic experiments, 18 µl mouse serum, buffered with 150 mM phosphate (Na_2HPO_4/NaH_2PO_4) to ensure a pH of 7.4, were mixed with a 500 mM solution of **3** in water. After incubation at 37°C for 24 hours, the platinum species were extracted using the established method described above.

Cyclic voltammetry

About 2 mg of the compound were dissolved in DMF (+ 0.1 M [*n*-Bu_4N][BF_4]) to obtain a final concentration of 1 mM. The measurements were conducted on an EG&G PARC 273A potentiostat/galvanostat with a scan rate of 100 mV/s. Nitrogen was bubbled through the solution to remove oxygen. A glassy carbon electrode was used as working electrode, which was polished before every measurement. The reference electrode was Ag/AgCl and the auxiliary electrode was a platinum wire. The potentials were referenced to an internal standard redox couple of ferrocenium/ferrocene ($E_{1/2}^{ox}$= +0.72 V vs. NHE).[9] The measurements were repeated three times and the mean values were calculated.

Reduction experiments

Phosphate buffer (250 mM, pH 7.4) containing 1 mM platinum compound with the addition of 10 eq. L-ascorbic acid was incubated at 20°C. The reaction was monitored on a Thermo Scientific Dionex UltiMate 3000 UHPLC-system using a Waters Acquity UPLC HSS T3 column. Milli-Q water, containing 0.1% formic acid, and acetonitrile containing 0.1% formic acid were used as eluents. For compounds **2**, **3**, **3a** and **3b,** a gradient of 1-40% acetonitrile over 5 min was used. For **1,** an isocratic gradient of 1% acetonitrile was used. For evaluating the current state of the reaction, the peak area of the parental complex was used. This was done due to the fact, that in most cases the reduction products did not have a high enough retention time to be distinguished from the injection peak.

Determination of pK_a values

About 2 mg of the compound were dissolved in 0.5 ml of D_2O, which resulted in a pD value of about 7. Afterwards a ^{1}H-NMR spectrum was recorded. Following this, the pD was adjusted with DNO_3 and a new spectrum was recorded. This was done in pD-increments of about 0.5 down to pD 1. After the spectrum at the lowest pD value was recorded, the pD was raised to basic conditions using NaOD, for validation of a reversible protonation. For determining the pK_a, the

ppm-shift of the acetato-ligand CH_3 signal in relation to the H_2O solvent peak was plotted against the pD. After fitting of a sigmoidal function, the pK_a could be determined.

For compound **3b**, the measurements were repeated in the presence of tetramethylammonium chloride as an internal standard resulting in a similar pK_a value.

Computational models

Two computational levels were used: I) optimization using B3LYP functional with 6-31+G(d,p) basis set and II) Stuttgart-Dresden pseudopotentials for the Pt atom. Water solution was simulated by COSMO implicit solvent and cavities based on Klamt atomic radii. For higher accuracy the same functional was combined with triple-zeta basis set (6-311++G(2df,2pd) with consistent extension of platinum pseudo orbitals[10] and D-PCM implicit solvation model with scaled-UAKS cavities.[11] For evaluation of Gibb's free energies Wertz corrections were utilized.[12,13]

X-ray

The X-ray intensity data were measured on Bruker X8 Apex2 diffractometer equipped with multilayer monochromators, Mo K/α INCOATEC micro focus sealed tubes and Cryoflex cooling system. The structure was solved by *heavy atoms method* and refined by *full-matrix least-squares techniques*. Non-hydrogen atoms were refined with *anisotropic displacement parameters*. The following software was used: *Bruker SAINT software package*[14] using a narrow-frame algorithm for frame integration, *SADABS*[15] for absorption correction, *OLEX2*[16] for structure solution, refinement, molecular diagrams and graphical user-interface, *Shelxle*[17] for refinement and graphical user-interface *SHELXS-2016*[18] for structure solution, *SHELXL-2016*[19] for refinement. Experimental data and CCDC-Codes (Available online: http://www.ccdc.cam.ac.uk/conts/retrieving.html) can be found in Table S2. Crystal data, data collection parameters, and structure refinement details are in Tables S3 and S4. Crystal structure visualized in Figure S8, packing in Figure S9.

Biological Methods

Cell culture conditions

The cancer cell models, RKO (human), HCT-116 (human) and CT26 (murine) were obtained from the American Tissue Culture Collection. RKO and HCT-116 cells were cultured in McCoy's

5a Medium (from Sigma-Aldrich, MO, USA), while CT26 cells were grown in Dulbecco´s modified eagle´s medium (DMEM)/F12 medium (1:1 from Sigma). Cells were maintained in medium containing 10% fetal calf serum (FCS, PAA, Linz, Austria) at 37.0 °C in a 5 % carbon dioxide-humidified atmosphere.

Cell viability assays

Cells were plated (3-4 x 10^3 cells/well) in 96-well plates and allowed to recover for 24 h. Then, cells were treated with **3**, **3a** or **3b** in concentrations ranging from 3.1 µM to 200 µM. After 72 h exposure, cell viability was measured by the 3-(4,5-dimethylthiazol-2-yl)-2,5-diphenyltetrazolium bromide (MTT)-based vitality assay (EZ4U; Biomedica, Vienna, Austria) according to the manufacturer's guidelines. GraphPad Prism software was used to calculate cell viability expressed as IC_{50} values calculated from full dose-response curves.

Cell uptake studies

To determine the cellular accumulation of the platinum complexes **3**, **3a** and **3b** in HCT116 and CT26 cells, they were incubated with 25 µM for 3 h at 37 °C, washed twice with PBS and the air dried cells lysed with 500 µL HNO_3 at room temperature for 1 h. Finally, 400 µl of the obtained lysates were dissolved in 7.6 mL aqua bidest and platinum levels were determined using ICP-MS. The used ICP-MS Agilent 7800® (Agilent Technologies, Tokyo, Japan) was equipped with an Agilent SPS 4 autosampler (Agilent Technologies, Tokyo, Japan) and a MicroMist nebulizer at a sample uptake rate of approx. 0.2 ml/min. The Agilent MassHunter® software package (Workstation Software, version C.01.04, Build 544.17, Patch 3, 2018) was used for data processing. The experimental parameters for ICP-MS are summarized in the table below. The instrument was tuned on a daily basis to achieve maximum sensitivity.

	**ICP-MS Agilent 7800**
**RF power (W)**	1550
**Cone material**	Nickel
**Carrier gas (L/min)**	1.07-1.09
**Plasma gas (L/min)**	15
**Monitored isotopes**	^{185}Re, ^{195}Pt, ^{196}Pt
**Integration time [s]**	1
**Number of sweeps**	100
**Number of replicates**	10

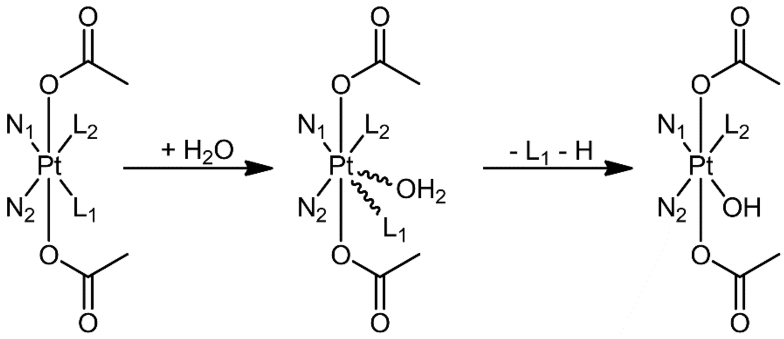

Scheme S1. Analyzed reaction steps by DFT calculations

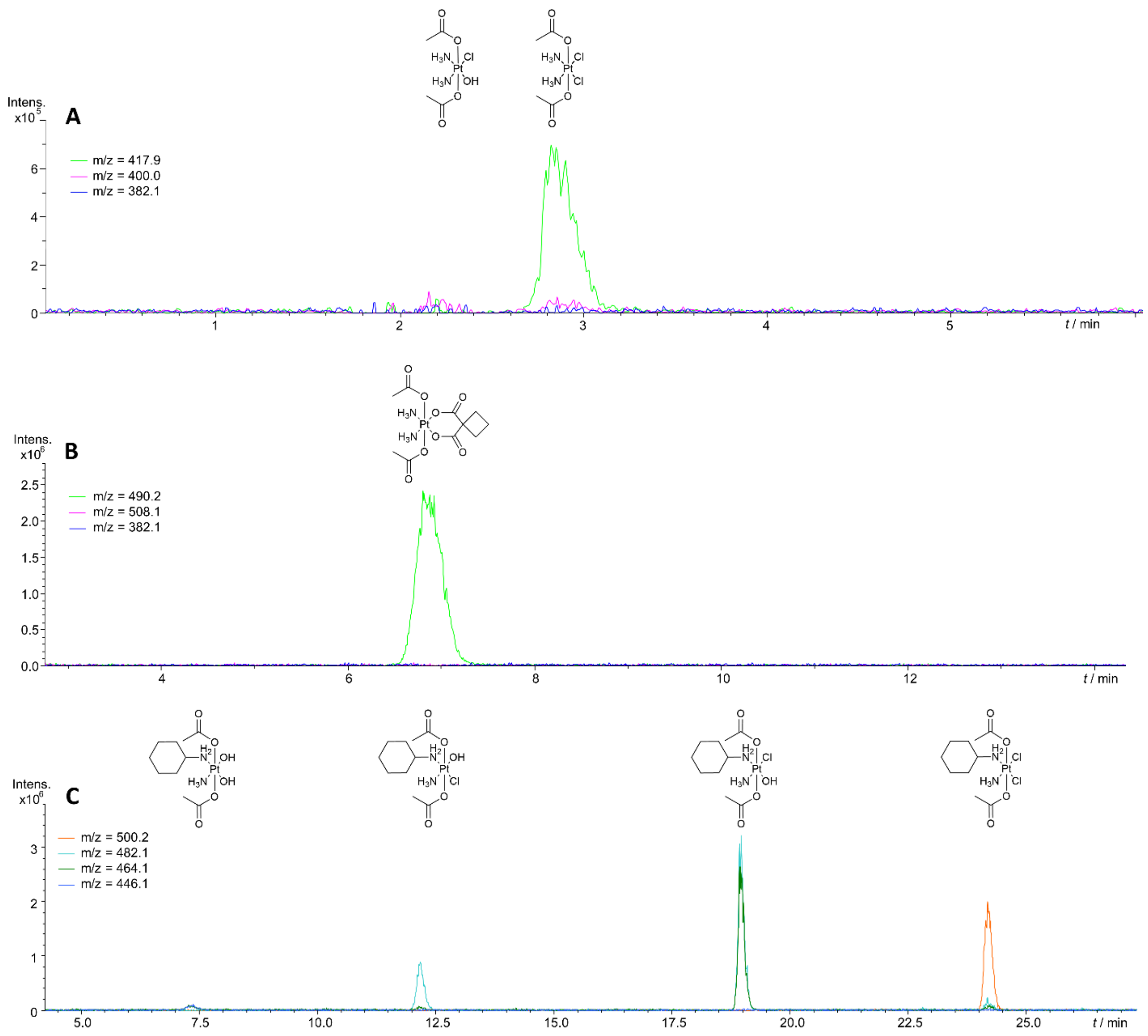

*Figure S1. HPLC-MS chromatograms of **A**) **1**, **B**) **2** and **C**) **4** after 24 h incubation in phosphate buffer (50 mM, pH=7.4) at 37°C.*

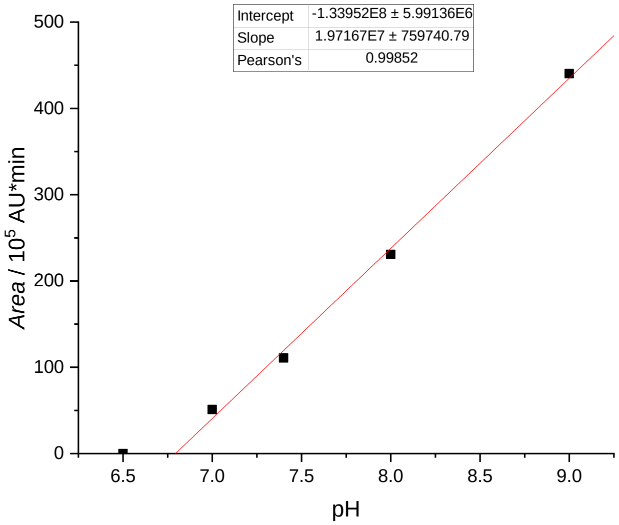

Figure S2. Correlation between pH and peak area of the formation of ***3b*** *after 24h incubation of* ***3*** *at 37°C in PB.*

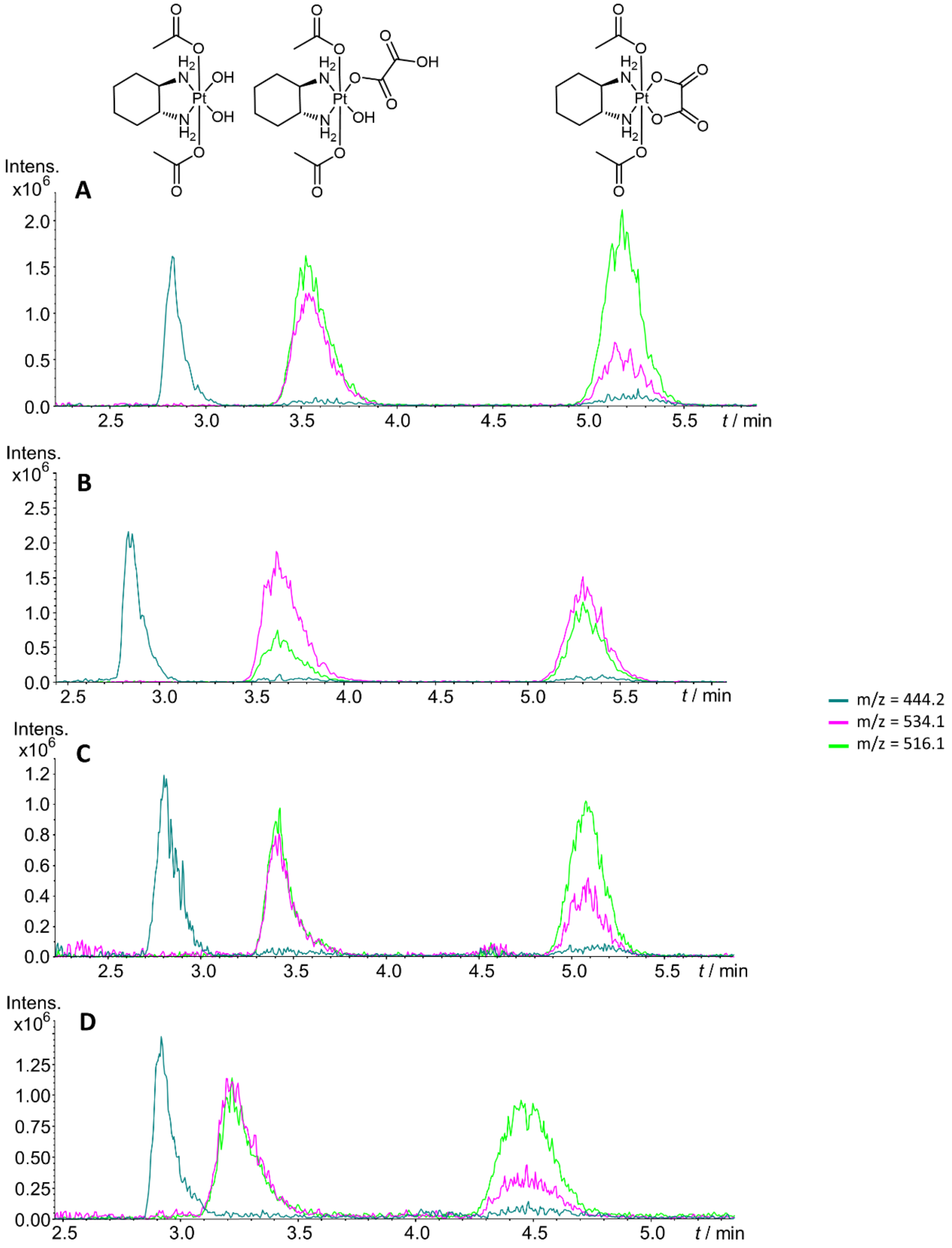

*Figure S3. Comparison of the hydrolysis products after 24 h incubation of **3** under different conditions at 37°C: **A**) 50 mM phosphate buffer, **B**) 50 mM ammonium carbonate buffer, **C**) RPMI-1640 with addition of 150 mM phosphate, **D**) mouse serum with addition of 150 mM phosphate. In the latter the shifts are different due to the extraction of the platinum compounds with methanol and the therefore higher amount of organic solvent in the sample.*

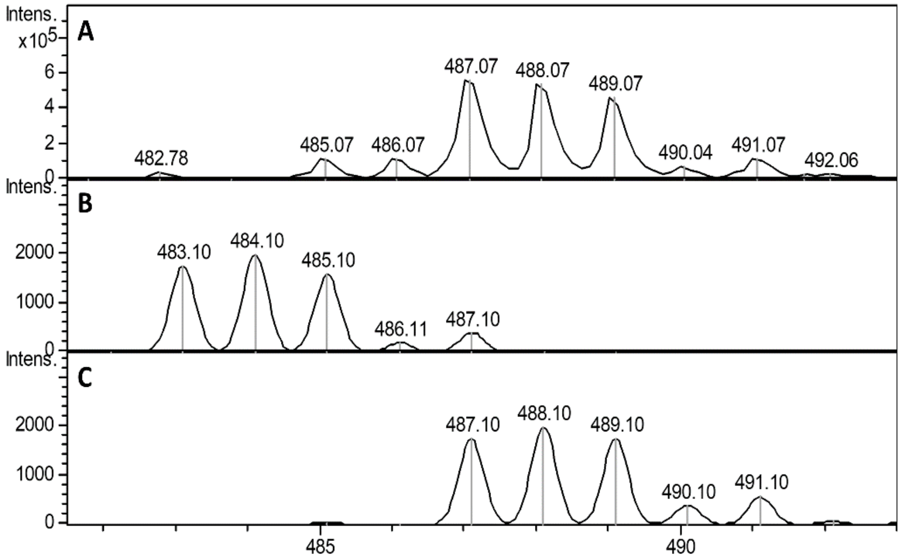

Figure S4. ***A***) ESI-MS measurement of ***3*** in $H_2{}^{18}O$ after 24 h, proving that solely ctc-[Pt(dach)(OAc)$_2$(^{18}OH)$_2$]+Na$^+$ is formed, ***B***) simulated ESI-MS spectrum of ctc-[Pt(dach)(OAc)$_2$(^{16}OH)$_2$]+Na$^+$, ***C***) simulated ESI-MS spectrum of ctc-[Pt(dach)(OAc)$_2$(^{18}OH)$_2$]+Na$^+$.

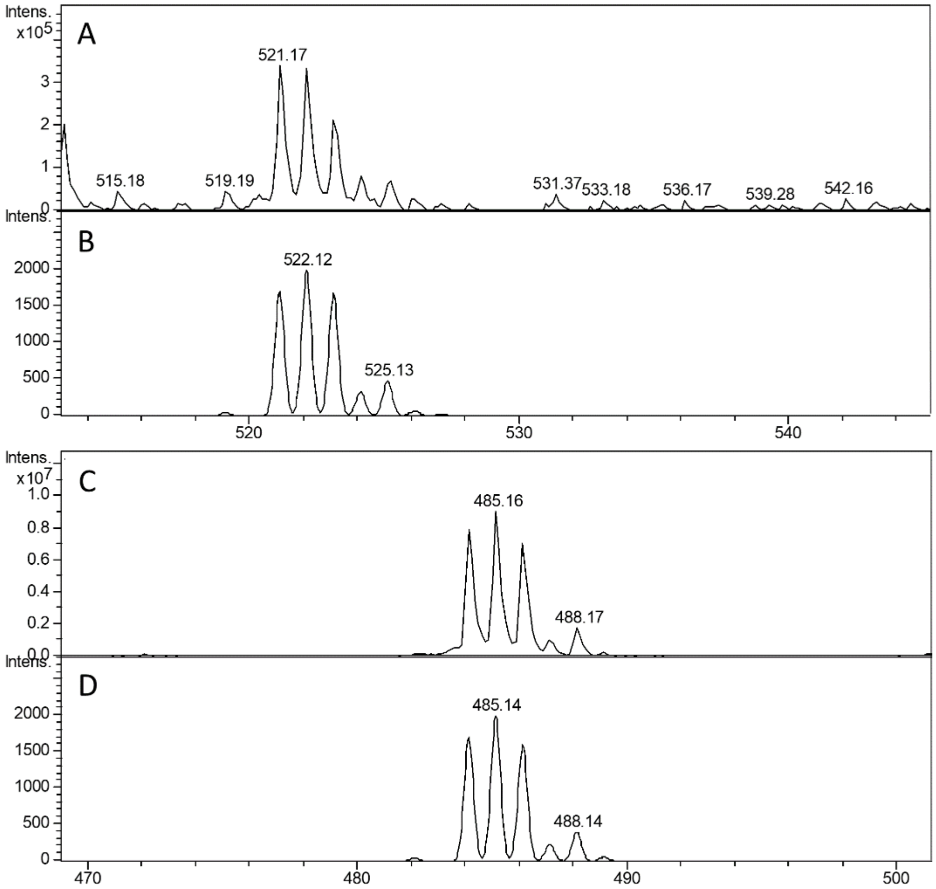

Figure S5. ***A***) ESI-MS measurement of ***3b*** in DMSO after 1 h showing the ctc-[Pt(DACH)(OAc)$_2$(OH)(dmso)]$^+$ adduct, ***B***) simulated ESI-MS spectrum of ctc-[Pt(DACH)(OAc)$_2$(OH)(dmso)]$^+$, ***C***) ESI-MS measurement of ***3b*** in ACN after 1 h showing the ctc-[Pt(DACH)(OAc)$_2$(OH)(acn)]$^+$ adduct, ***D***) simulated ESI-MS spectrum of ctc-[Pt(DACH)(OAc)$_2$(OH)(acn)]$^+$

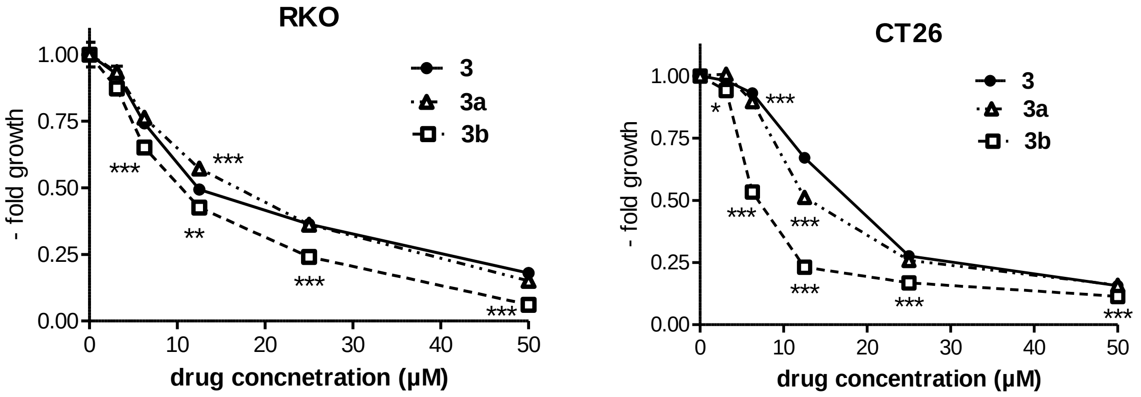

*Figure S6. Anticancer activity in cell culture. Human RKO and murine CT26 cells were treated with the indicated concentrations of **3**, **3a** and **3b** for 72 h. The values given are means and standard deviations (SD) of one representative experiment performed in triplicates. * p<0.05, *** p<0.001, significantly different to control, calculated by Two-Way ANOVA and Bonferroni post-test.*

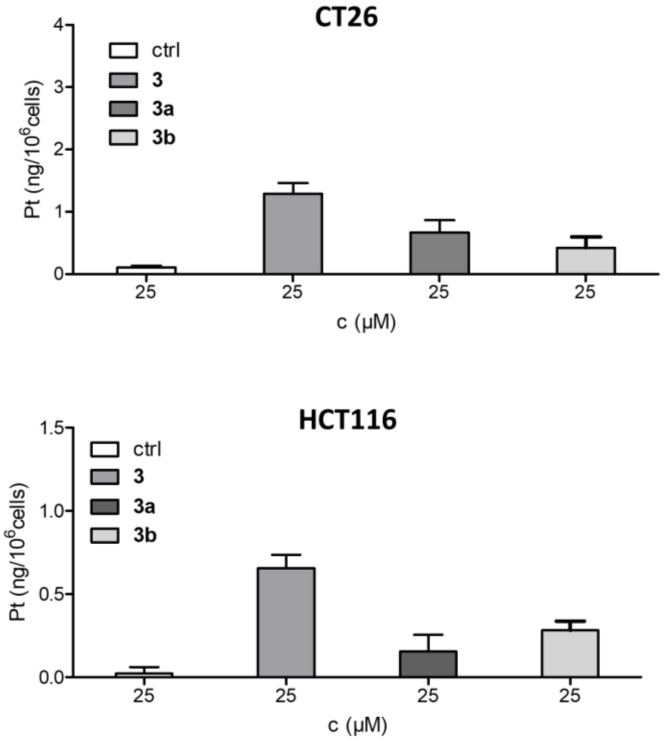

*Figure S7. Cellular drug uptake. Murine CT26 and human HCT116 cells were treated with the indicated concentration of **3**, **3a** and **3b** for 3 h. The values given are means and standard deviations (SD) of one representative experiment performed in triplicates. The differences of the platinum uptake of the complexes is not significant (p<0.05, calculated by Two-Way ANOVA and Bonferroni post-test).*

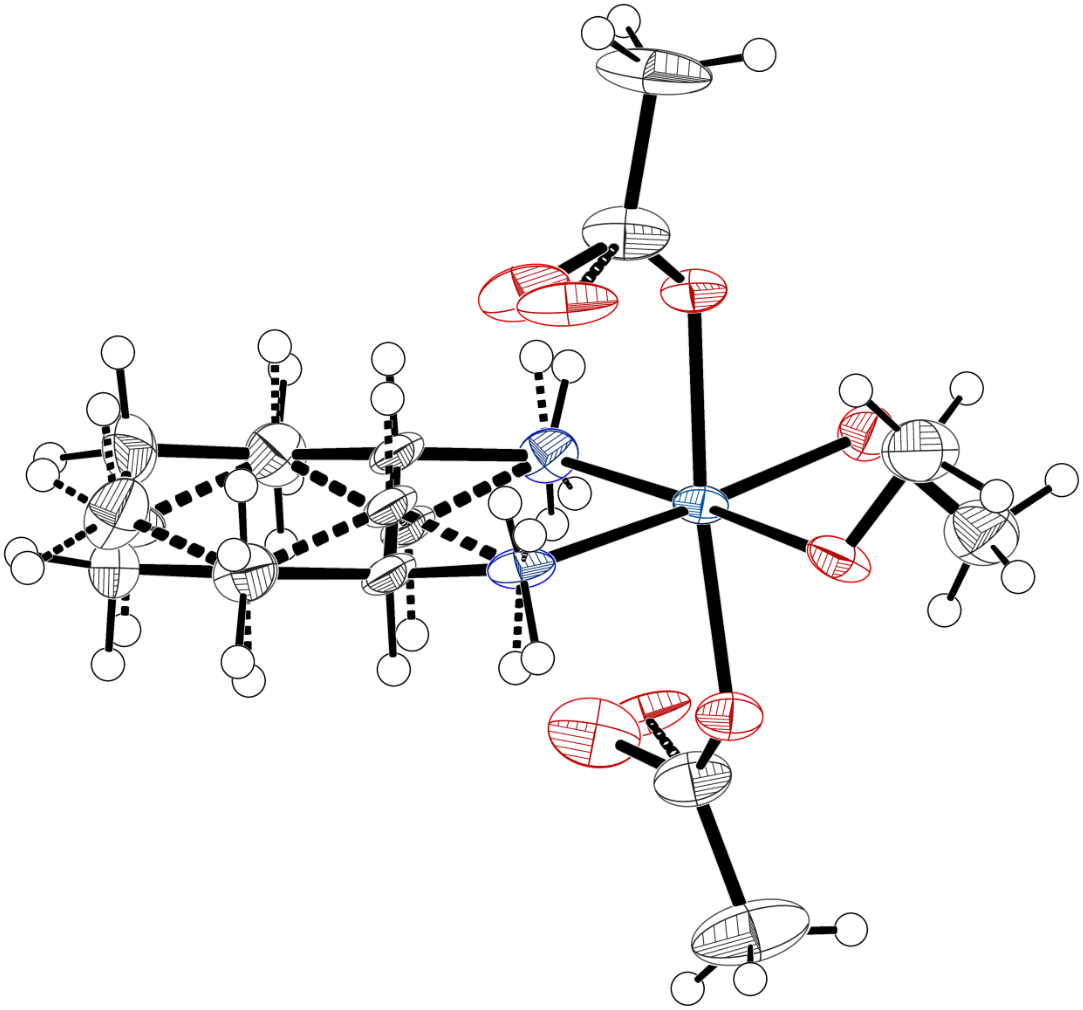

Figure S8. Asymmetric Unit of CCDC-1890270.Anisotropic displacement ellipsoids visualized with 50% probability. Bond precision: C-C = 0.0165Å. The degree of main residue disorder is 29%, forced by disorder of cyclohexane-1,2-diamine racemate as 1R,2R and 1S,2S. The molecule is characterized by two moderate intramolecular hydrogen bonds (two by each enantiomer) located between diamines and acetates.

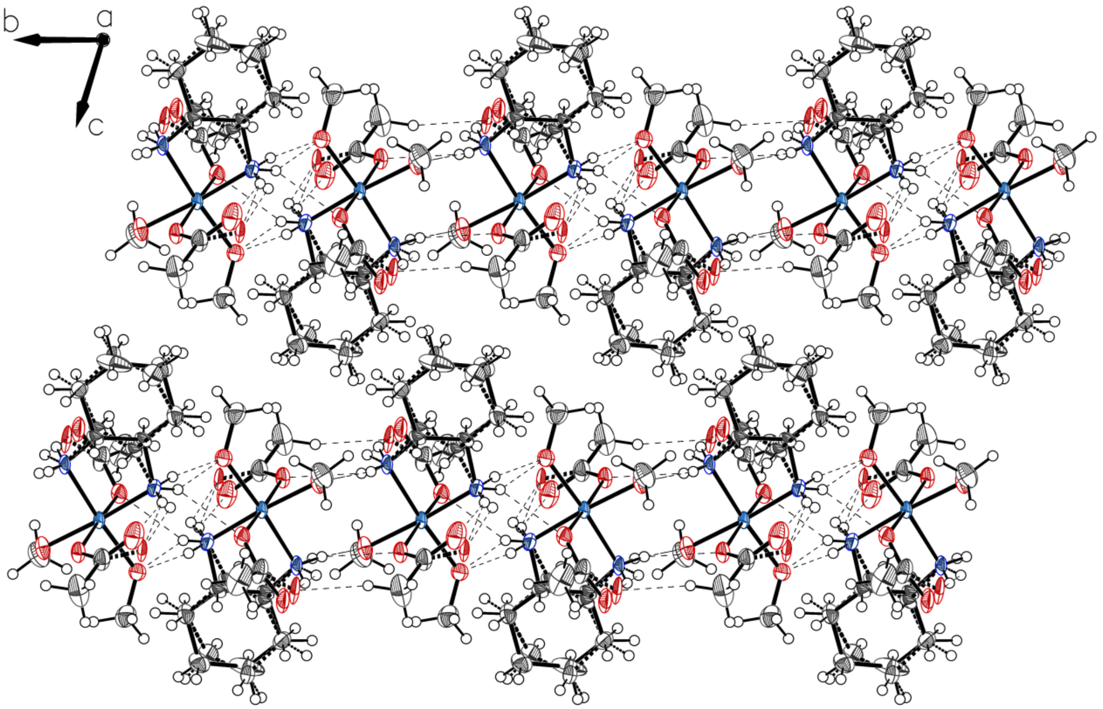

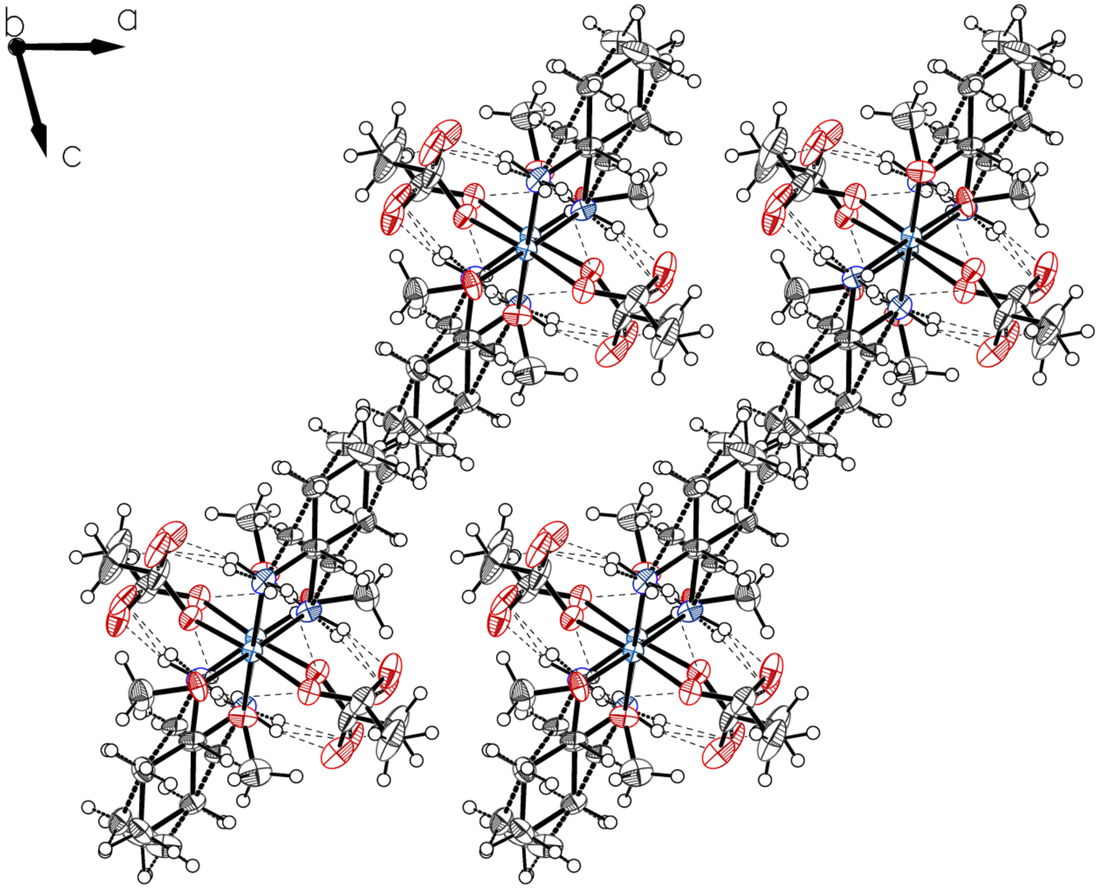

Figure S9. Packing along axis a and b. Intermolecular weak and moderate hydrogen bonds along axis b characterize the packing of CCDC-1890270.

*Table S1. Binding energies of the Pt(IV) ligands in kcal/mol (ac = acetate; N1, N2 = amino ligand(s); LL = leaving ligand(s); X_h = monohydroxido form of the complexes **1**-**7**).*

**Compound**	BE(ac1)	BE(ac2)	BE(N1)	BE(N2)	BE(LL1)	BE(LL2)
**1**	-70.1	-70.1	-65.2	-65.2	-54.4	-54.4
**1_h**	-68.7	-70.9	-62.9	-59.8	-49.9	-81.7
**2**	-72.8	-70.7	-67.7	-69.3	-149.7	
**2_h**	-67.9	-60.2	-57.4	-63.9	-68.2	-81.4
**3**	-74.2	-74.2	-147.7		-133.7	
**3_h**	-65.9	-69.3	-132.4		-59.8	-75.2
**4**	-70.3	-70.4	-67.2	-73.7	-53.4	-51.8
**4_h1**	-67.9	-69.7	-60.2	-70.2	-48.1	-80.5
**4_h2**	-67.9	-70.4	-63.7	-66.4	-49.8	-77.4
**5**	-69.7	-69.7	-149.4		-51.8	-51.8
**5_h**	-68.3	-66.9	-139.2		-48.9	-76.9
**6**	-69.9	-69.9	-146.7		-52.9	-52.9
**6_h**	-68.9	-67.3	-136.7		-49.5	-78.5
**7**	-74.8	-74.8	-72.4	-72.4	-140.1	
**7_h**	-65.9	-73.1	-66.8	-63.6	-63.0	-78.4

Table S2. Experimental parameter of CCDC-1890270.

**Machine**	**Source**	**Temp.**	**Detector Distance**	**Time/ Frame**	**#Frames**	**Frame width**
Bruker		[K]	[mm]	[s]		[°]
X8	Mo	100	35	45	2007	0.6

Table S3. Sample and crystal data of CCDC-1890270.

**Chemical formula**	C12H26N2O6Pt	**Crystal system**	triclinic	
**Formula weight [g/mol]**	489.44	**Space group**	*P-1*	
**Temperature [K]**	100	**Z**	2	
**Measurement method**	\f and \w scans	**Volume [Å^3]**	849.0(4)	
**Radiation (Wavelength [Å])**	MoKα (λ = 0.71073)	**Unit cell dimensions [Å] and [°]**	9.512(2)	67.633(8)
**Crystal size / [mm^3]**	0.1 × 0.02 × 0.02		10.127(3)	69.222(8)
**Crystal habit**	clear colourless needle		10.514(3)	69.717(8)
**Density (calculated) / [g/cm^3]**	1.915	**Absorption coefficient / [mm^{-1}]**	8.29	
**Abs. correction Tmin**	0.7452	**Abs. correction Tmax**	0.5904	
**Abs. correction type**	multiscan	**F(000) [e$^-$]**	476	

Table S4. Data collection and structure refinement of CCDC-1890270

**Index ranges**	-11 ≤ h ≤ 11, -12 ≤ k ≤ 12, -12 ≤ l ≤ 12	**Theta range for data collection [°]**	4.334 to 51.424	
**Reflections number**	21793	**Data / restraints / parameters**	3175/18/230	
**Refinement method**	Least squares	**Final R indices**	all data	R1 = 0.0455, wR2 = 0.0806
**Function minimized**	Σ w(F_o^2 - F_c^2)^2		I>2σ(I)	R1 = 0.0359, wR2 = 0.0769
**Goodness-of-fit on F^2**	1.144	**Weighting scheme**	w=1/[$\sigma^2(F_o^2)+(0.0345P)^2+0.7025P$]	
**Largest diff. peak and hole [e Å^{-3}]**	2.17/-1.65		where $P=(F_o^2+2F_c^2)/3$	

Table S5. Bond lengths of CCDC-1890270.

Atom	Atom	Length/Å	Atom	Atom	Length/Å
Pt1	O1	2.007(5)	C3	C2B	1.518(19)
Pt1	O2	2.000(5)	C3	C4B	1.47(3)
Pt1	O3	2.001(5)	C6	C1A	1.456(18)
Pt1	O4	2.005(5)	C6	C5A	1.50(2)
Pt1	N1	2.043(6)	C6	C1B	1.618(17)
Pt1	N2	2.044(6)	C6	C5B	1.60(2)
O1	C7	1.301(9)	C7	C8	1.499(11)
O2	C9	1.291(9)	C7	O5A	1.235(10)
O3	C11	1.379(9)	C7	O5B	1.231(10)
O4	C12	1.417(9)	C9	C10	1.494(11)
N1	C1A	1.495(17)	C9	O6A	1.234(10)
N1	C1B	1.511(18)	C9	O6B	1.230(10)
N2	C2A	1.516(19)	C1A	C2A	1.50(3)
N2	C2B	1.458(17)	C4A	C5A	1.49(5)
C3	C2A	1.511(18)	C1B	C2B	1.43(3)
C3	C4A	1.57(5)	C4B	C5B	1.46(4)

Table S6.Bond angles of CCDC-1890270.

Atom	Atom	Atom	Angle/˚	Atom	Atom	Atom	Angle/˚
O1	Pt1	N1	86.6(2)	O1	C7	C8	112.8(7)
O1	Pt1	N2	96.8(2)	O5A	C7	O1	124.5(18)
O2	Pt1	O1	174.32(19)	O5A	C7	C8	120.8(18)
O2	Pt1	O3	81.6(2)	O5B	C7	O1	124.7(17)
O2	Pt1	O4	94.5(2)	O5B	C7	C8	121.3(17)
O2	Pt1	N1	97.6(2)	O2	C9	C10	113.0(7)
O2	Pt1	N2	87.5(2)	O6A	C9	O2	123.4(15)
O3	Pt1	O1	94.5(2)	O6A	C9	C10	121.6(16)
O3	Pt1	O4	91.9(2)	O6B	C9	O2	124.8(13)
O3	Pt1	N1	175.88(19)	O6B	C9	C10	120.3(13)
O3	Pt1	N2	92.2(2)	N1	C1A	C2A	107.4(12)
O4	Pt1	O1	81.4(2)	C6	C1A	N1	116.0(13)
O4	Pt1	N1	92.2(2)	C6	C1A	C2A	113.7(14)
O4	Pt1	N2	175.69(19)	C3	C2A	N2	112.6(13)
N1	Pt1	N2	83.7(2)	C1A	C2A	N2	110.2(14)
C7	O1	Pt1	126.7(5)	C1A	C2A	C3	112.1(12)
C9	O2	Pt1	126.6(5)	C5A	C4A	C3	113(2)
C11	O3	Pt1	119.4(5)	C4A	C5A	C6	112(2)
C12	O4	Pt1	118.0(5)	N1	C1B	C6	106.1(11)
C1A	N1	Pt1	110.8(7)	C2B	C1B	N1	111.4(14)
C1B	N1	Pt1	104.6(7)	C2B	C1B	C6	112.8(12)
C2A	N2	Pt1	108.9(7)	N2	C2B	C3	115.5(12)
C2B	N2	Pt1	108.7(8)	C1B	C2B	N2	105.8(13)
C2A	C3	C4A	109.3(19)	C1B	C2B	C3	110.3(15)
C4B	C3	C2B	113.8(15)	C5B	C4B	C3	113(2)
C1A	C6	C5A	111.6(13)	C4B	C5B	C6	113.4(19)
C5B	C6	C1B	105.5(12)				

[1] S. C. Dhara, *Indian J Chem* **1970**, *8*, 193.

[2] C. R. Kowol, P. Heffeter, W. Berger, B. K. Keppler, J. Mayr, V. Pichler, **2017**, WO 2017097986.

[3] Y. Kidani, K. Inagaki, M. Iigo, A. Hoshi, K. Kuretani, *J Med Chem* **1978**, *21*, 1315.

[4] M. J. Abrams, et al., *Pt(IV) Complexes*, **1994**, 0 328 274 B1.

[5] M. Ravera, E. Gabano, I. Zanellato, D. Osella, *Dalt Trans* **2016**, *45*, 5300.

[6] R. Kizu, T. Nakanishi, M. Miyazaki, Y. Kidani, *Anti-Cancer Drugs* **1996**, *7*, 248.

[7] S. R. A. Khan, S. Huang, S. Shamsuddin, A. R. Khokhar, *Bioorg Med Chem* **2000**, *8*, 515.

[8] C. K. J. Chen, J. Z. Zhang, J. B. Aitken, T. W. Hambley, *J Med Chem* **2013**, *56*, 8757.

[9] W. C. Barrette, H. Johnson, D. T. Sawyer, Anal Chem 1984, 56, 1890.

[10] J. V. Burda, M. Zeizinger, J. Šponer, J. Leszczynski, *J Chem Phys* **2000**, *113*, 2224.

[11] T. Zimmermann, J. V. Burda, *Dalt Trans* **2010**, *39*, 1295.

[12] F. Šebesta, J. V. Burda, *Eur J Inorg Chem* **2018**, *2018*, 1481.

[13] M. J. Cheng, R. J. Nielsen, W. A. Goddard, *Chem Commun* **2014**, *50*, 10994.

[14] Bruker SAINT v8.38A Copyright © 2005-2018 Bruker AXS

[15] Sheldrick, G. M. (1996). SADABS. University of Göttingen, Germany.

[16] Dolomanov, O.V., Bourhis, L.J., Gildea, R.J, Howard, J.A.K. & Puschmann, H. , OLEX2, *J Appl Cryst* **2009**, *42*, 339

[17] C. B. Huebschle, G. M. Sheldrick and B. Dittrich, ShelXle: a Qt graphical user interface for SHELXL, *J Appl Cryst* **2011,** *44*, 1281

[18] Sheldrick, G. M. **2015**. SHELXS v 2016/4. University of Göttingen, Germany.

[19] Sheldrick, G. M. **2015**. SHELXL v 2016/4. University of Göttingen, Germany.
